# Initial validation of the LSPain scale: a behaviour-based clinical tool for canine lumbosacral pain

**DOI:** 10.3389/fvets.2025.1684577

**Published:** 2025-10-15

**Authors:** Roger Medina-Serra, Sandra Sanchis-Mora, Clara Conde Ruiz, Francesco Aprea, Eliseo Belda, Francisco G. Laredo, Miguel Ángel Cabezas

**Affiliations:** ^1^Anaesthesia and Pain Management, North Downs Specialist Referrals, Part of Linnaeus Veterinary Limited, Bletchingley, United Kingdom; ^2^Escuela Internacional de Doctorado de la Universidad de Murcia, Programa en Ciencias Veterinarias, Universidad de Murcia, Murcia, Spain; ^3^Blaise Veterinary Referral Hospital, Birmingham, United Kingdom; ^4^Service d’anesthésie et algologie, CHV AniCura Armonia, Vaulx-Milieu, France; ^5^Hospital Veterinari Canis Mallorca, Mallorca, Spain; ^6^Departamento de Medicina y Cirugía Animal, Facultad de Veterinaria, Universidad de Murcia, Murcia, Spain; ^7^Hospital Veterinario Universidad de Murcia, Murcia, Spain; ^8^Departamento de Medicina y Cirugía Animal, Universidad Complutense de Madrid, Madrid, Spain

**Keywords:** lumbosacral pain, pain assessment, pain scale, canine degenerative lumbosacral stenosis (CDLSS), dog

## Abstract

**Introduction:**

This study aimed to validate the LSPain Scale, a novel 4-point ordinal instrument for assessing lumbosacral pain in dogs based on behavioural responses to palpation of the lumbosacral region.

**Methods:**

A preliminary analysis was conducted using clinical data to inform sample size estimation and to explore the scale’s convergent validity and responsiveness. Subsequently, a prospective validation was performed using 50 anonymised clinical videos retrospectively collected from client-owned dogs evaluated for suspected lumbosacral pain. Dogs represented a broad clinical spectrum and were graded using the LSPain Scale, where scores range from 0 (no pain) to 3 (severe pain). Trained observers, including pain specialists and general practitioners, independently scored the videos while blinded to clinical data.

**Results:**

Psychometric evaluation demonstrated strong convergent and discriminant validity, responsiveness to treatment, inter- and intra-rater reliability, and usability across experience levels.

**Discussion:**

The findings support the LSPain Scale as a valid and reliable clinician-based tool for standardised assessment and monitoring of lumbosacral pain in dogs.

## 1 Introduction

Lumbosacral (LS) pain is a frequent clinical presentation in dogs, particularly in older and large-breed individuals, where spinal pathologies such as intervertebral disc herniation, foraminal stenosis, and radiculopathy are common (1). Because animals cannot self-report pain and caregiver assessments are inherently subjective and variable, validated and standardised tools are essential to reliably evaluate pain in veterinary patients (2). However, clinical assessment of LS spinal pain remains inconsistent due to the absence of specific, validated tools (2, 3). This gap has been formally recognised by international expert panels, including the 2017 (2, 3) and 2025 Pain in Animals Workshop (PAW) reports, which identified the development of condition-specific clinical metrology instruments (CMIs) and observer-based tools as a key priority in veterinary pain research.

Both caregiver-reported and clinician-based assessments have been used to evaluate LS pain in dogs. However, these approaches are often non-standardised and lack formal validation (4, 5). The Canine Brief Pain Inventory (CBPI), although originally validated for osteoarthritis and bone cancer (6, 7), has nonetheless been applied in studies involving degenerative intervertebral disc disease (8) and LS pain (9), serving as a proxy to assess pain severity and its impact on function in dogs. This cross-application highlights a pressing need for dedicated tools that specifically capture clinically relevant behaviours associated with LS pain in dogs.

The 2025 PAW report further emphasises the importance of selecting outcome measures aligned with study objectives, acknowledging that different instruments evaluate distinct dimensions of the pain experience. This supports the development and formal validation of condition-specific scales such as the LSPain Scale, which aims to quantify pain severity in dogs with LS pathology using structured behavioural criteria observed during palpation.

Psychometric validation is essential to ensure that a clinical instrument provides accurate, consistent, and clinically relevant information (10, 11). Psychometric properties of behavioural and observer-based pain scales typically include content, convergent, and discriminant validity, as well as inter- and intra- rater reliability (10–19).

For a behavioural pain scale, content validity reflects whether the scale adequately represents the full range of behaviours relevant to the construct being measured, in this case being behavioural indicators of LS pain elicited during palpation. A scale with strong content validity includes relevant, representative items without including irrelevant components. In the absence of a true gold standard for pain assessment in animals (2), convergent validity is commonly used to support the validity of a scale by comparing it with other established pain measures (7, 15). While a true gold standard for pain is absent, criterion validity may still be assessed by comparing the scale with other validated pain measures (13). Thus, a pain scale with good convergent validity is expected to produce scores that vary meaningfully across individuals with differing levels of pain and align with other validated pain measures. In the context of our study, discriminant validity refers to the scale’s capacity to distinguish between dogs that are clinically painful and those that are not. Thus, a scale with good discriminant validity should yield significantly different scores between painful and non-painful groups. Responsiveness is the capacity of a scale to detect meaningful changes in the condition being measured. In the context of pain assessment in small animals, this property is typically demonstrated by a meaningful decrease in scores following an analgesic intervention (14). Reliability refers to the consistency and reproducibility of the scale. Inter-rater reliability assesses the extent to which different observers assign similar scores when evaluating the same subject, while intra-rater reliability reflects the consistency of scores assigned by the same observer on repeated occasions. Good reliability ensures that the scale produces stable results under consistent conditions, irrespective of who performs the evaluation or when it is applied (10).

The aim of this study was to evaluate the psychometric properties of the LSPain Scale, a novel 4-point ordinal instrument developed to assess LS pain in dogs. Specific objectives were to evaluate its convergent and discriminant validity, responsiveness to treatment, and inter- and intra-rater reliability. We hypothesised that the LSPain Scale would demonstrate the necessary psychometric properties to support its initial validation as a clinician-based tool for the assessment of LS pain in dogs.

## 2 Materials and methods

This was a prospective, observer-blinded validation study using retrospectively collected, anonymised video recordings of dogs undergoing LS palpation during routine clinical evaluations at North Downs Specialist Referrals. Ethical approval was granted by the Association of Veterinary Anaesthetists (AVA) Ethical Review Group (2025-008).

Video selection was performed by a single clinician (RMS), who developed and routinely applied the LSPain Scale in clinical practice. Video recordings were reviewed and retrieved retrospectively until the target number or recordings was reached. Recordings had been acquired during consultations either using a camera mounted on a tripod or filmed by an assistant. Only video recordings that met predefined inclusion criteria were considered. These criteria required: (1) adequate visibility of the dog’s posture and behaviour during LS palpation; (2) absence of caregiver-identifiable information; and (3) no verbal commentary on pain status during the recording. Dogs with concomitant hip pathology were excluded to minimise diagnostic confounding during behavioural evaluation. Dogs were included regardless of their ongoing medication status or behavioural presentation at the time of consultation.

Caregivers provided both verbal and informed written consent (Appendix 1) for the use of anonymised clinical data and videos for clinical research and publication purposes. Recordings were obtained during routine clinical evaluations to support behavioural assessment in dogs presenting with suspected LS pain. All videos were anonymised prior to use, and no identifying information was included.

### 2.1 Scale development

The LSPain Scale was developed by a board-certified anaesthetist and pain clinician (RMS) drawing on clinical experience in assessing acute and chronic pain in dogs. The scale captures behavioural responses to palpation using standardised criteria across four ordinal grades (0–3) (Table 1).

**Table 1:** LSPain Scale: 4-point ordinal scale for assessing lumbosacral pain in dogs based on behavioural responses to palpation.

Grade	Pain level	Behavioural description
0	No Pain	The patient remains indifferent and shows no signs of discomfort during palpation.
1	Mild Pain	Subtle shifting during palpation indicating slight discomfort, without vocalisation or significant avoidance.
2	Moderate Pain	Flinching, soft whimpering, or intentional movement away. May show claudication, guarding, or mild stress behaviours (e.g., muscle tension).
3	Severe Pain	Strong avoidance responses such as abrupt movement away, loud vocalisation (yelping), or aggression (snapping/biting), with or without Grade 2 signs.

Scores range from 0 (no pain) to 3 (severe pain), reflecting increasing pain severity. Stress-related behaviours, such as aggression or withdrawal, occur only when lumbosacral pain is elicited during palpation.

### 2.2 Preliminary testing and sample size calculation

To inform the sample size and design of the prospective psychometric evaluation, a preliminary analysis was conducted to assess convergent validity and responsiveness of the LSPain Scale using retrospectively collected clinical data.

Preliminary convergent validity was explored using data from 18 dogs evaluated at two separate time points (baseline and post-analgesic intervention) and 23 dogs with single assessments. This yielded a total of 59 observations. At each assessment, pain was scored using the LSPain Scale by a single ECVAA anaesthetist blinded to caregiver-reported outcomes (RMS), and caregivers completed the Canine Brief Pain Inventory (CBPI), from which the pain severity score (PSS) was extracted for analysis. To minimise diagnostic confounding during this preliminary validation, dogs with concurrent hip pathology were excluded, given the frequent coexistence of hip and LS disease and the clinical overlap between these conditions (1).

The LSPain Scale was developed to grade pain expression during clinical examination, without aiming to determine the anatomical source of pain. Considering the frequent coexistence of hip and LS pathology in dogs and the overlap in their clinical signs (1), accurate localisation of the primary pain generator remains a challenge. Furthermore, as reported in our previous MRI-based study, LS pain may still be present in a subset of dogs without detectable structural abnormalities, highlighting the limitations of imaging-based diagnosis in certain cases (1).

The association between clinician-assigned ordinal scores and caregiver-derived PSS (treated as a continuous variable) was analysed using Spearman’s rank correlation, which demonstrated a statistically significant strong positive correlation (correlation coefficient of 0.618, *p* < 0.001). Based on these findings, a minimum of 18 dogs was calculated to be sufficient to detect a correlation coefficient of 0.618 with 80% power and a two-sided alpha of 0.05.

Responsiveness was evaluated in a subset of 18 dogs with paired video assessments. Pain management, which included pharmacological adjustments and/or interventional pain management procedures, was tailored to the clinical needs of each patient. Pain was scored at each time point using the LSPain Scale by a single ECVAA anaesthetist blinded to caregiver-reported outcomes (RMS). To assess the ability of the scale to detect clinically meaningful change, a Wilcoxon signed-rank test was performed on pre- and post-treatment scores. The analysis demonstrated a statistically significant reduction in LSPain Scale scores following analgesic intervention (*V* = 171, *p* = 0.00014), supporting the scale’s sensitivity to change in pain status over time.

The mean LSPain Scale score decreased from 2.56 pre-treatment to 0.39 post-treatment, reflecting a substantial improvement in clinical condition. The estimated effect size (Cohen’s *r* = 0.899) was classified as large, further confirming that the change observed was not only statistically significant but also likely to be clinically significant. These findings provide preliminary evidence that the LSPain Scale is responsive to analgesic intervention, an essential psychometric property for any pain assessment tool intended for monitoring treatment efficacy in clinical practice. To support planning of the prospective phase, a sample size calculation based on this effect size was performed using a paired t-test approximation. A minimum of 8 paired observations per observer was estimated to provide 80% power to detect a similar treatment effect at a two-sided significance level of 0.05.

For intra-rater reliability, a sample size of 24 videos was estimated to be sufficient to detect a weighted Cohen’s kappa of 0.70, assuming a null hypothesis value of 0.40, *α* = 0.05, and 80% power. For inter-rater reliability assessment, 47 videos were estimated to be sufficient to detect an intraclass correlation coefficient (ICC[2,k]) of 0.75, assuming a null hypothesis value of 0.40, *α* = 0.05, 80% power, and three raters.

To meet these sample size requirements and ensure adequate representation across all four ordinal pain grades, a target sample size of 50 videos was selected for the full-scale validation study.

### 2.3 Observer recruitment

For the prospective validation of the LSPain Scale, a dual-panel approach was adopted to assess the scale’s performance across varying levels of clinical expertise. The expert group (EG) consisted of board-certified anaesthetists with established experience in the assessment and management of canine LS pain. The generalist group (GG) included veterinary surgeons working in primary care practice, to assess the usability and interpretability of the LSPain Scale in a general clinical setting.

All observers were blinded to patient identity, diagnosis, treatment status, and time point of assessment. Prior to scoring, observers underwent training and familiarisation with the scale, which included a review of the LSPain Scale definitions, one example video for each pain grade, and user guidance. This training aimed to harmonise interpretation of behavioural responses across observers. Observers were instructed to have the LSPain Scale available during scoring to ensure consistent and standardised application of the grading criteria.

### 2.4 Sampling strategy and data handling

Eligible videos were identified and trimmed by a single operator (RMS) to isolate 10–20 s clips focused on LS palpation and the associated behavioural responses. Corresponding caregiver-completed CBPI questionnaires were scanned and paired with each video.

To ensure broad clinical applicability of the scale, video selection was based on the LSPain Scale scores assigned during the original clinical evaluation, ensuring representation across all four pain grades (Grade 0 to 3). Observers were blinded to this sampling strategy. Grade 0 videos included both healthy controls and clinical cases with confirmed LS pathology that were deemed non-painful on examination. This approach aimed to enhance the generalisability of the scale by ensuring applicability across a range of clinical scenarios, including pain-free dogs with and without spinal pathology. To minimise diagnostic confounding, dogs with concurrent hip pathology were excluded prior to video selection, consistent with the preliminary phase.

To support assessment of responsiveness, some dogs were included at multiple clinical time points where pain status differed (before and after analgesic intervention). In a few cases, dogs were represented more than once with equivalent pain status to prevent recognition bias and discourage observers from making relative comparisons. Each video was treated as an independent observation.

Once video selection was completed, all data was transferred to a second investigator (MAC), who anonymised the dataset. The anonymised videos were randomised,^1^ and a secure online platform was developed to facilitate observer access. Each observer received a private link to the platform, where they viewed and scored the anonymised videos. Scores were submitted directly to MAC, who compiled the results into an Excel spreadsheet. Statistical analyses were performed using R (RStudio, version 2024.12.0 + 467) by two investigators (RMS and MAC).

### 2.5 Validation framework and statistical analysis

The validation framework was designed to assess the psychometric properties of the LSPain Scale through independent evaluation by both expert and generalist observer panels.

Convergent validity was assessed using Spearman’s rank correlation coefficient (*ρ*) to evaluate the association between the median LSPain Scale score per dog (aggregated across expert observers) and the corresponding caregiver-reported pain severity score (PSS) from the Canine Brief Pain Inventory (CBPI).

Discriminant validity was evaluated by comparing the aggregated median LSPain Scale scores between dogs without observable pain behaviours (Grade 0, including healthy controls and non-painful clinical cases) and those exhibiting pain-related behaviours (Grades 1 to 3). Dogs were classified as non-painful only if the median score assigned by observers within a group was 0; any dog with a median score above 0 was classified as painful. Group differences were assessed using the Mann–Whitney U test, and effect size was calculated using Cohen’s *r* to aid clinical interpretation. This analysis was performed separately for each observer group.

Responsiveness was assessed in a subset of dogs with paired recordings obtained before and after analgesic intervention. For each dog and time point, the median LSPain Scale score across expert observers was calculated. The Wilcoxon signed-rank test was applied to compare pre- and post-treatment scores, and Cohen’s r was used to estimate the effect size and its clinical relevance.

Inter-rater reliability was assessed separately for expert and generalist groups using raw (non-aggregated) ordinal LSPain Scale scores to preserve inter-observer variation. Agreement was quantified using weighted Cohen’s kappa for all pairwise comparisons, Krippendorff’s alpha to assess overall consistency, and the intraclass correlation coefficient (ICC[2,k]) to estimate absolute agreement amongst all observers. These metrics were computed using the raw (non-aggregated) ordinal data to preserve inter-individual variability.

Finally, intra-rater reliability was assessed within the expert group by comparing scores assigned during two separate scoring sessions. Agreement for each observer was evaluated using weighted Cohen’s kappa, with randomised video order between sessions to reduce recall bias.

Reliability was interpreted according to the following thresholds: values <0.5 were considered poor, 0.5–0.75 moderate, >0.75–0.9 good, and >0.90 excellent (20).

All statistical analyses were conducted using R (R Studio, version 2024.12.0 + 467). Non-parametric methods were employed as appropriate to accommodate the ordinal nature of the LSPain Scales. For group-level analyses of convergent validity, discriminant validity, and responsiveness, individual scores assigned by expert observers were aggregated per dog using the median. A two-sided *p*-value of <0.05 was considered statistically significant for all analyses.

## 3 Results

Based on the preliminary analysis, a dual-panel design was implemented, including ten veterinarians in the generalist group and three board-certified anaesthetists in the expert group, to enable assessment of both inter- and intra-rater reliability.

Amongst the ten veterinary surgeons in the generalist group, seven were Members of the Royal College of Veterinary Surgeons (MRCVS) and actively practising in the United Kingdom, and the remaining three participants were based in Argentina, Portugal, and Spain, respectively. All generalist observers completed a single scoring session. The expert group consisted of three ECVAA-certified anaesthetists (SS, CC, FA) with long-standing experience in managing chronic pain, each of whom completed two scoring sessions, separated by a two-week interval, with video order re-randomised between sessions.

The final dataset consisted of fifty videos derived from thirty-seven individual dogs. Distribution across the four pain grades of the LSPain Scale was as follows: eighteen videos were classified as Grade 0 (no pain), of which eleven were clinical cases deemed non-painful on examination and seven were obtained from healthy control dogs. Ten videos were classified as Grade 1 (mild pain), fifteen as Grade 2 (moderate pain), and seven as Grade 3 (severe pain). Thirteen dogs were represented more than once in the dataset. Eight of these contributed paired videos obtained before and after analgesic treatment, which were used to evaluate responsiveness. The remaining five dogs contributed repeated videos showing the same pain grade to minimise recognition bias and support inter-rater reliability assessment. Each video was treated as an independent observation.

### 3.1 Convergent validity

**Figure 1:**
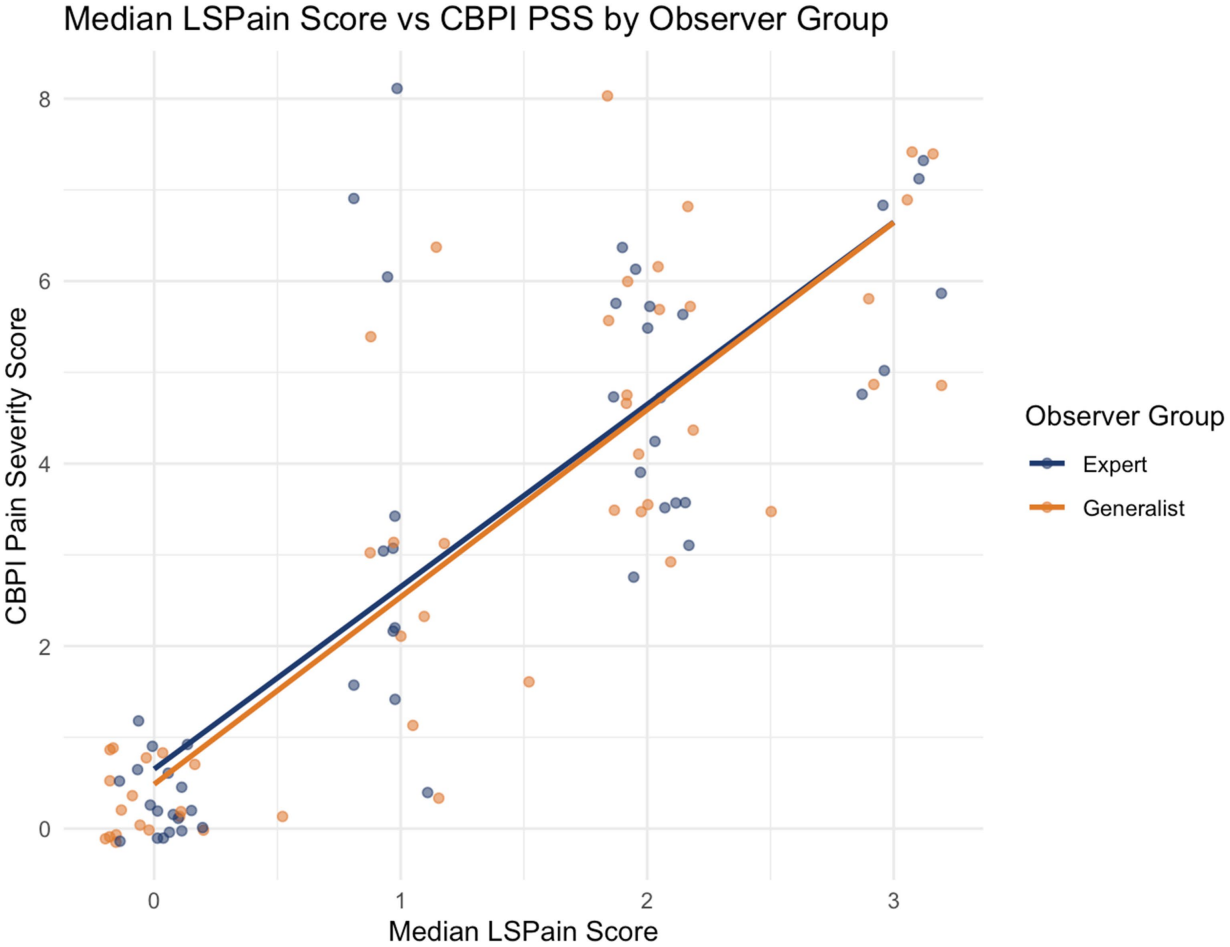
Scatter plot showing the relationship between the median LSPain score and the CBPI Pain Severity Score (PSS) for both expert (navy blue) and generalist (orange) observer groups. Each point represents a video recording assessed using the LSPain Scale, with the median score across observers plotted against the corresponding caregiver-reported CBPI PSS for that case. Linear regression lines illustrate the strength and direction of association within each observer group. While greater dispersion was observed at LSPain Grade 1, the overall association remains very strong for both groups (*ρ* = 0.838 and *ρ* = 0.856 for expert and generalist groups, respectively; *p* < 0.001), providing strong evidence of convergent validity between clinician-assigned LSPain scores and caregiver-reported pain severity.

A very strong positive correlation was found for both the expert (*ρ* = 0.838, *p* < 0.001) and the generalist (*ρ* = 0.856, *p* < 0.001) groups, supporting that clinician-assigned LSPain scores accurately reflect caregiver-perceived pain severity in dogs with LS pain (Figure 1).

### 3.2 Discriminant validity

**Figure 2:**
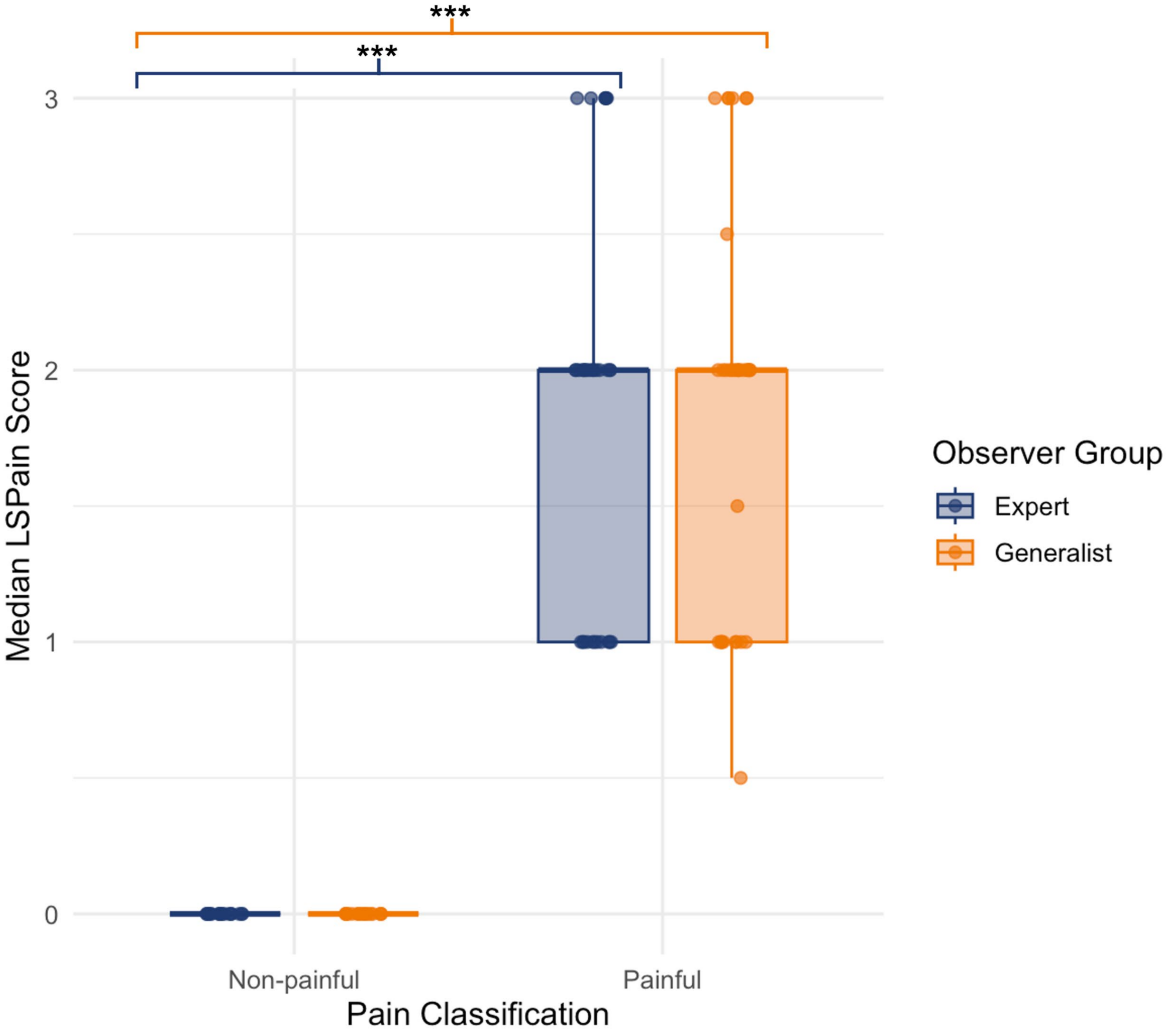
Boxplots showing aggregated median LSPain scores for dogs classified as non-painful or painful, based on assessments by expert (navy blue) and generalist (orange) observer groups. Each point represents the median score per video. Boxplots display the interquartile range and median, with overlaid jittered points illustrating score variability. Statistically significant differences were found between non-painful and painful dogs in both observer groups, supporting the discriminant validity of the LSPain Scale (*p < 0.05, **p < 0.01, ***p < 0.001).

In the expert group, aggregated LSPain scores were significantly higher in dogs classified as painful (*n* = 32) compared to those classified as non-painful (*n* = 18) (Wilcoxon rank-sum test: *W* = 0, *p* < 0.001), with a large effect size (Cohen’s *r* = 0.859). In the generalist group, the same pattern was observed, with significantly higher scores in painful (*n* = 33) than non-painful (*n* = 17) dogs (*W* = 0, *p* < 0.001; Cohen’s *r* = 0.843). The corresponding score distributions for each group are illustrated in Figure 2. These results support the discriminant validity of the LSPain Scale across both observer groups.

### 3.3 Responsiveness

**Figure 3:**
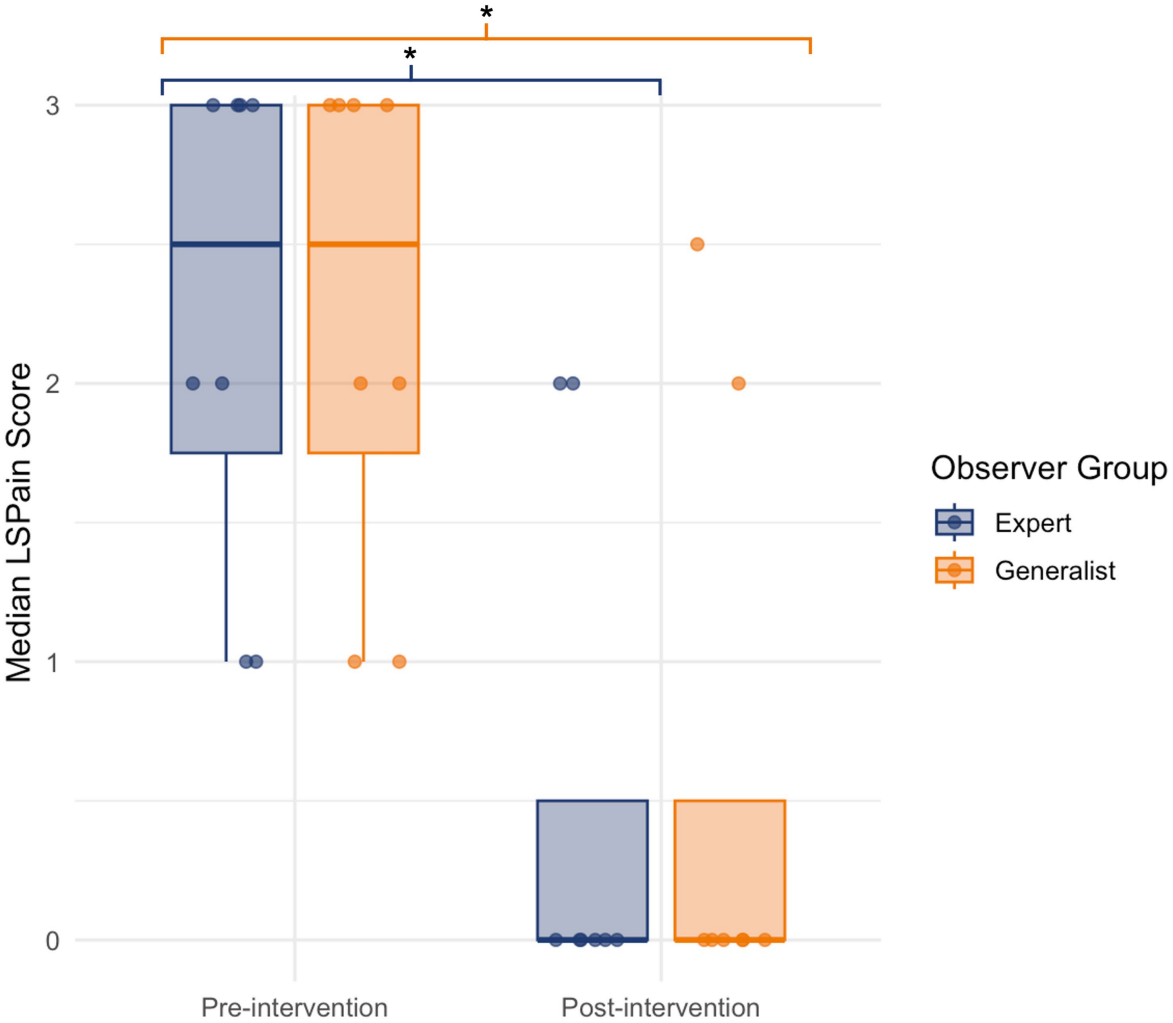
Boxplot showing median LSPain scores pre- and post-intervention for expert (navy blue) and generalist (orange) observer groups. Each point represents the aggregated median score per dog, based on paired video assessments. Boxplots display the interquartile range and median, with jittered points overlaid to illustrate score distribution. Both groups demonstrated significantly lower scores following intervention, supporting the responsiveness of the LSPain Scale (*p < 0.05, **p < 0.01, ***p < 0.001).

In the expert group, a statistically significant reduction in aggregated median LSPain scores was observed following analgesic intervention in the subset of dogs with paired pre- and post-treatment recordings (*n* = 8) (Wilcoxon signed-rank test: *V* = 28.0, *p* = 0.021). The effect size was large (Cohen’s *r* = 0.819), with a mean reduction of 1.75 points. In the generalist group, a similar pattern was observed. Median LSPain scores were significantly lower following treatment (Wilcoxon signed-rank test: *V* = 33.5, *p* = 0.034), with a large effect size (Cohen’s *r* = 0.748) and a mean reduction of 1.69 points. The corresponding pre- and post-intervention score distributions for both groups are presented in Figure 3. These findings support the responsiveness of the LSPain Scale to detect clinically relevant changes in pain following treatment, when applied by both expert and generalist observers.

### 3.4 Inter-rater reliability

**Figure 4:**
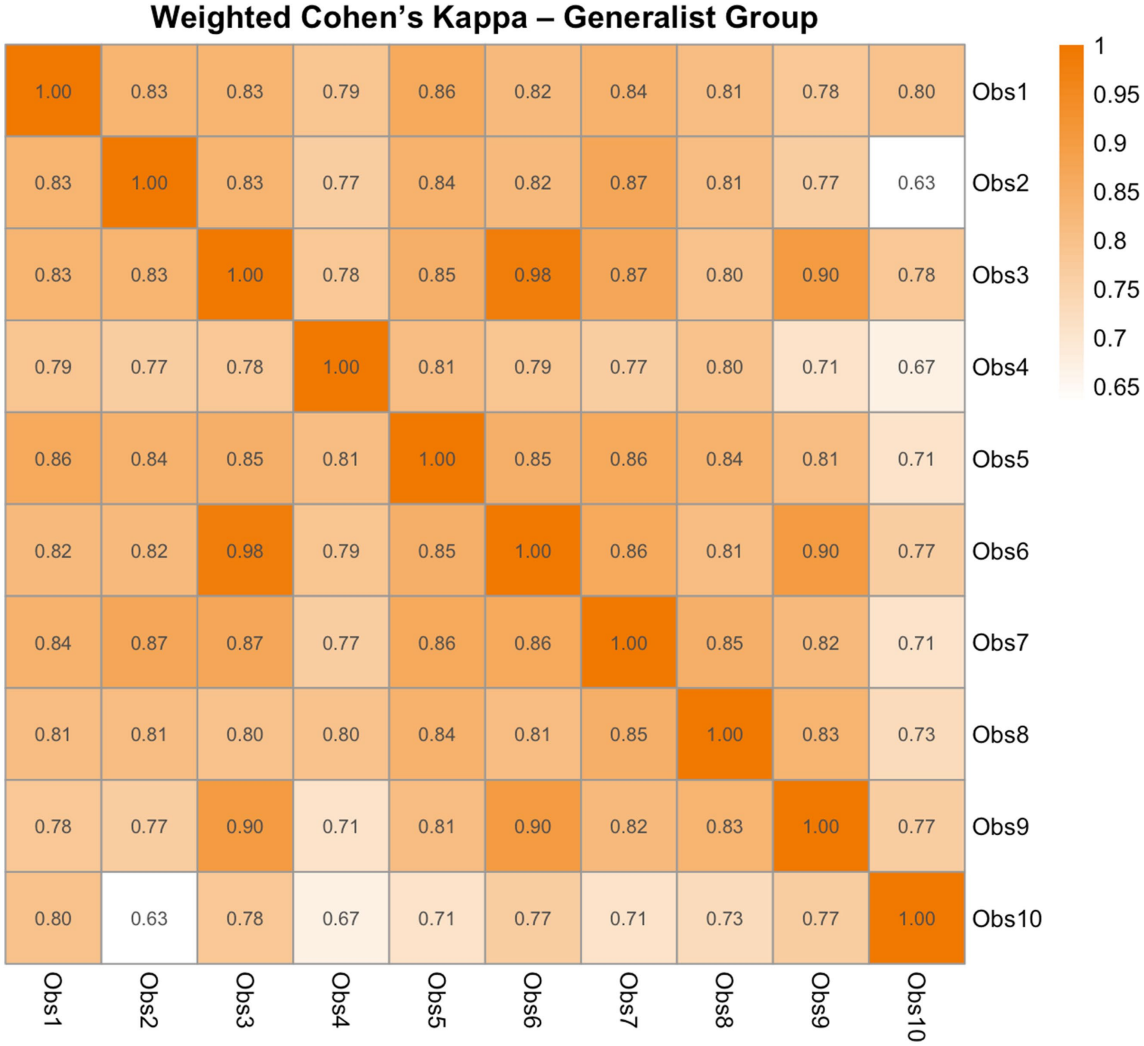
Heatmap of pairwise weighted Cohen’s kappa coefficients showing inter-rater agreement amongst the 10 generalist observers of the LSPain Scale. Each cell represents the level of agreement between two observers, with values ranging from 0 (no agreement) to 1 (perfect agreement). Quadratic weights were applied to account for the ordinal nature of the LSPain scores. Warmer colours indicate stronger agreement.

In the expert group, pairwise agreement via weighted Cohen’s kappa indicated good agreement between Expert 1 and Expert 2 (*κ* = 0.731) and between Expert 2 and Expert 3 (*κ* = 0.678), and excellent agreement between Expert 1 and Expert 3 (*κ* = 0.882). All comparisons were statistically significant (*p* < 0.001). Group-level consistency was good, with Krippendorff’s alpha of 0.787. The ICC[2,k] for absolute agreement amongst experts was 0.911 [95% CI: 0.835–0.951; *F*(49, 98) = 13.82, *p* < 0.0001], indicating excellent inter-rater reliability. In the generalist group, pairwise kappa values ranged from 0.632 to 0.981 (full representation in Figure 4), representing moderate to excellent agreement, with most comparisons falling in the good to excellent range. All were statistically significant (*p* < 0.001). Krippendorff’s alpha was 0.812, indicating good consistency at the group level. The ICC[2,k] was 0.977 [95% CI: 0.966–0.986; *F*(49, 441) = 47.51, *p* < 0.0001], reflecting excellent absolute agreement. Together, these findings confirm that the LSPain Scale demonstrates good to excellent inter-rater reliability across both expert and generalist observers.

### 3.5 Intra-rater reliability

Expert 1 demonstrated excellent intra-rater reliability (*κ* = 0.914, *z* = 6.5, *p* < 0.0001). Expert 2 showed good agreement (*κ* = 0.81, *z* = 5.96, *p* < 0.0001), while Expert 3 also achieved excellent reliability (*κ* = 0.975, *z* = 6.92, *p* < 0.0001). These findings indicate consistency in how observers applied the LSPain Scale across repeated assessments.

## 4 Discussion

This study presents the initial psychometric evaluation of the LSPain Scale, a clinician-based instrument designed to quantify LS pain in dogs based on behavioural responses elicited during palpation of the LS region. Developed to address the current lack of validated, condition-specific tools for LS pain assessment in dogs (2, 3), the LSPain Scale demonstrated strong converting and discriminant validity, responsiveness, and reliability in both specialist and generalist observers.

While debates persist regarding the objectivity of pain assessment in non-verbal species, experts reaffirm the value of structured, observer-based approaches in both clinical and research settings (2, 21). The inability of veterinary patients to communicate their pain verbally (22) parallels situations in humans, where behavioural pain scales are routinely applied to non-verbal patients such as neonates and individuals with advanced dementia, with tools like the Pain Assessment in Advanced Dementia (PAINAD) Scale (23). In the assessment of acute pain in small animals, both clinician- and caregiver-based evaluations are considered important (12, 13, 15, 17, 19, 24, 25). Clinician-based assessment is particularly prominent in the postoperative period, where pain expression can be closely monitored by the attending team, and caregiver access to the hospital environment is limited or absent. In contrast, the clinical evaluation of chronic pain in dogs continues to rely almost exclusively on caregiver-reported instruments, as pain-associated behaviours indicative of pain are primarily expressed at the home environment (3). Nonetheless, clinical examination remains an essential component of chronic pain assessment, offering valuable insights not obtainable from caregiver-reported data alone (23). The Canine Osteoarthritis Staging Tool (COAST) initiative has highlighted the need for integrated assessment strategies (26, 27), emphasising that clinical examination is indispensable for comprehensive staging and management of chronic pain.

The LSPain Scale represents, to our knowledge, the first clinician-based instrument specifically developed for LS pain in dogs to undergo initial psychometric validation. Its aim is not to replace existing caregiver-based tools, but rather to complement them, enriching the clinical evaluation of patients with LS pain and supporting more tailored and responsive pain management throughout the course of the condition.

Convergent validity was evaluated with the association between clinician-assigned LSPain scores and caregiver-perceived CBPI-PSS. The CBPI was originally developed for the assessment of chronic pain in dogs with osteoarthritis (6) and has undergone validation for both osteoarthritis and bone cancer (6, 7). In the absence of a gold standard for pain assessment in dogs, construct validity of the CBPI was supported through multiple complementary approaches, including factor analysis of its internal structure, as well as assessments of discriminant and convergent validity, responsiveness, and reliability. Since then, the CBPI has been widely adopted in studies investigating spinal and neuropathic pain in dogs, including LS conditions (8, 9). Its broad application across diverse clinical contexts and its translation into multiple languages (28, 29) reflects its acceptance as a general instrument for assessing chronic pain burden in veterinary patients. The use of the CBPI as a reference measure for convergent validity has precedent in veterinary pain research (19), and its use in the present study was similarly a pragmatic necessity due to the lack of a validated gold standard for assessing LS pain in dogs. This limitation highlights a critical gap in the field and reinforces the need for condition-specific tools such as the LSPain Scale, which represents an initial step towards standardising LS spinal pain assessment in dogs. Preliminary analysis supported the convergent validity of the LSPain Scale, which was further confirmed in the prospective phase through consistent associations between clinician-assigned scores and caregiver-reported pain severity across both expert and generalist observers.

Discriminant validity was supported by statistically significant differences in LSPain Scale scores between dogs with (Grades 1–3) or without (Grade 0) observable pain-related behaviours. This finding was consistent across both expert and generalist observers. Although the non-painful group included some clinical cases deemed pain-free on examination, the presence of undetected or subclinical pain in these dogs could have attenuated between-group differences. Nevertheless, clear score separation was still evident for both groups, supporting the scale’s ability to distinguish between dogs that do or do not exhibit pain-associated behaviours during clinical examination.

Responsiveness was supported by a reduction in LSPain Scale scores following analgesic management in the subset of dogs with paired video assessments. This was observed in both the preliminary analysis and the prospective phase across expert and generalist groups, indicating that the scale was sensitive to changes in pain-associated behaviours after treatment. These findings support the use of the scale for monitoring response to analgesic intervention in dogs with LS pain.

Analgesic interventions were not standardised, reflecting the clinical nature of the study and being tailored to each patient’s needs (detailed in Supplementary material). In the preliminary analysis (*n* = 18), all dogs underwent interventional pain management procedures, with two also receiving adjustments to their oral analgesic regimen. In the prospective phase (*n* = 8), five dogs received interventional pain management and three had adjustments to their oral medication. The greater mean reduction in scores observed in the preliminary analysis compared with the prospective phase may be partly explained by the higher proportion of dogs undergoing interventional pain management in the former cohort, as these procedures may be associated with greater analgesic effects. Furthermore, since all dogs in this study were referred cases already receiving analgesic medication, adjustments to an existing regimen may have a smaller impact on pain than initiating treatment in patients not previously receiving analgesia. Although the lack of standardisation in analgesic interventions may have introduced variability and is acknowledged as a potential limitation, it also increases the relevance of the findings to everyday clinical practice. Dogs with naturally occurring LS pain are rarely managed with a single drug. In most cases, treatment involves combinations of pharmacological agents and, in some instances, advanced modalities such as interventional pain management. Evaluating the scale in this context is therefore likely to provide a more applicable assessment of its performance in real-world settings than testing it solely against the effect of one analgesic treatment.

Establishing a clinically meaningful change threshold is essential for interpreting the responsiveness of pain assessment instruments. In the present study, responsiveness of the LSPain Scale was supported by a statistically and clinically significant reduction in scores following analgesic intervention, as observed in both the preliminary and prospective analyses. In the preliminary cohort (*n* = 18), the mean reduction in LSPain scores post-treatment was 2.17 points. In the prospective phase (*n* = 8), expert and generalist observers recorded mean reductions of 1.75 and 1.69 points, respectively. Based on these findings, a two-point reduction on the LSPain Scale is proposed as a clinically meaningful threshold for treatment response. Although smaller changes may reflect improvement, the four-point ordinal structure precludes intermediate values and limits resolution. A two-point change offers a pragmatic balance between clinical relevance and interpretability, while reducing the risk of false positives when evaluating treatment effects.

It is important not to equate responsiveness with the need for treatment. In patients with mild pain (Grade 1), a two-grade improvement would not be measurable, as this would require a reduction to a score below zero. In such cases, clinical decision-making should not be delayed until a two-grade reduction is possible. For dogs with mild but persistent pain over several weeks, intervention is advisable, and a one-grade improvement in these patients should be considered a clinically relevant response. This welfare consideration should not be used to purposefully inflate the apparent efficacy of a treatment modality in retrospective or prospective studies. Its purpose is solely to ensure that treatment is not withheld in patients with persistent mild pain and that a one-grade reduction from mild to no pain is recognised as clinically meaningful in this specific context.

The reliability of the LSPain Scale was assessed between (both groups) and within (expert group) observers to determine the consistency of its application across different raters and over time. Inter-rater agreement ranged from moderate to excellent, with good to excellent agreement at the group level. These findings indicate that veterinarians of different experience can apply the LSPain Scale in a reproducible manner, supporting its use in both clinical and research settings where multiple observers may be involved in pain assessment. Intra-rater agreement ranged from good to excellent, confirming that individual clinicians applied the scale consistently across repeated assessments. This supports the use of the LSPain Scale for monitoring pain trajectories in dogs during follow-up.

The LSPain Scale was purposefully designed with a simplified structure comprising four ordinal grades to enhance clinical applicability. This approach mirrors the rationale behind the development of the short-form Glasgow Composite Measure Pain Scale CMPS-SF (12), where reduction and restructuring of items improved usability in practice without undermining core psychometric properties. While the simplicity of the LSPain Scale facilitates consistent implementation in clinical settings, it inherently limits the detection of subtle variations within each pain category. This categorical approach was chosen to enhance ease of interpretation and favour clinical applicability.

In medicine, it is recognised that there is no gold standard in pain assessment, as each tool has strengths and limitations (16). Numerical rating scales (NRS) and visual analogue scales (VAS) are widely used and have demonstrated good validity across a range of clinical and research settings (16). However, numerical pain ratings are recognised as difficult to interpret in isolation due to the lack of intrinsic meaning (30, 31), and their clinical relevance improves when linked to structured categories that reflect pain severity and impact, such as those used in the revised Graded Chronic Pain Scale (32, 33). This approach is reflected in the International Classification of Diseases (ICD-11) for chronic pain developed by the International Association for the Study of Pain (IASP) (31). The ICD-11 includes extension codes for grading pain severity as mild, moderate, or severe, based on recommended but not validated NRS or VAS scores for pain intensity, pain-related distress, and interference with daily activities. A further example of the widespread use of categorical pain interpretation is found in the treatment guidelines for cancer pain by the World Health Organization (WHO), which are structured around pain intensity categories of mild, moderate, and severe (34). Numerous human studies have sought to define cut-off points for numerical pain ratings to facilitate a categorical translation (30, 35, 36). These efforts reflect a shared goal: to retain the sensitivity and analytical advantages of numerical scales while enabling categorical interpretation to support treatment decisions and improve communication. Nonetheless, identified thresholds may vary according to patient population, pain condition, and context, indicating that the relationship between numerical scores and perceived pain intensity is not fixed or linear (30, 35, 36). This variability further supports the value of categorical frameworks that classify pain in clinically meaningful terms. Considering the challenges associated with interpreting pain intensity from numerical scores alone, veterinary pain assessment tools for acute pain such as the CMPS-SF (12) and the FGS (13) have also introduced empirically derived cut-off values to assist clinical decision-making. These thresholds are not meant to categorise pain severity but to indicate when analgesic intervention may be appropriate. While such cut-offs enhance the practical utility of each tool, they are scale-specific and do not capture the broader context or continuum of pain beyond the intervention threshold. In contrast, categorical classifications may offer a more interpretable and user-friendly framework for assessing pain severity, particularly when prioritising treatment or communicating the significance of observed pain behaviours.

While observer-based pain scoring tools in veterinary medicine must be simple and clear (25), the role of observer training and familiarisation with the scales in ensuring their reliability remains a subject of debate. Various studies report training of the observers prior to scoring (13, 19, 37–40), with some supporting that training improves reliability (38, 41, 42), while others suggest the effect may be limited (37, 39). As more evidence emerges, inconsistencies in reliability have become increasingly apparent (39). In this context, observer training is gaining recognition as an important factor in the appropriate and consistent application of pain assessment tools (13, 38, 41, 42). In our study, no formal evaluation of training effects on outcomes was performed. Instead, we applied a structured process aimed at familiarising observers with the LSPain Scale and ensuring its adequate use. Given that observer involvement introduces inherent variability, familiarisation and training is unlikely to introduce harm and may contribute to improved scoring consistency.

While observer training is often considered less critical in the context of chronic pain, where the caregiver is typically regarded as the expert due to their familiarity with the animal’s normal behaviour (3), further research is needed to evaluate the impact of structured training on the reliability of observer-based pain assessment tools in veterinary settings.

In the present study, all observers underwent a structured training session before scoring. This included a video presentation outlining the LSPain Scale and explaining each pain grade in detail, accompanied by one illustrative video per grade. Observers were instructed to refer to the scale during evaluation to enhance consistency.

In addition to variability introduced during pain scoring, an important consideration when applying the LSPain Scale is the potential influence of differences in how the palpation stimulus is delivered. In this study, all examinations were performed by a single operator, ensuring a consistent and standardised approach to the clinical stimulus and supporting the validity of the psychometric assessment. However, in clinical practice, variation in technique across operators may affect the behavioural responses elicited and influence the scores assigned. Future studies involving multiple clinicians performing the examination would help elucidate the potential impact of inter-operator variability during clinical examination on the scale’s performance. At the pain management unit where the LSPain Scale was developed, the examiner aimed to apply moderate pressure during palpation, with the intent to elicit clinically meaningful responses without causing unnecessary distress. The rationale for incorporating palpation was to provoke behaviours that might not otherwise be expressed, as without such a stimulus the ability to assess pain severity during examination would be markedly reduced. In the author’s experience, excessive pressure may induce false-positive responses and undermine the primary diagnostic objective of confirming the LS region as a pain generator. A potential limitation relates to the inherent variability of behavioural response associated with breed and individual temperament. Although the sample size calculation was based on preliminary data and therefore inherently accounted for some degree of variability, the population studied may not fully represent the wide range of behavioural responses across breeds and individual dogs. Such variability could have influenced the results and warrants further exploration in future studies. An additional methodological consideration is that pain scoring in this study was conducted retrospectively via video review, rather than by the clinicians performing the physical examination. This design allowed for standardisation of the visual stimulus and blinding to clinical data but differs from typical clinical practice, where the examiner interprets responses in real-time with access to full patient history.

To support wider application of the LSPain Scale, a laminated sheet containing the LSPain Scale and a QR code linking to the training video has been provided in the Supplementary materials. This resource is intended as a practical guide for both clinical and research use it provides recommended guidance on how the clinical examination should be performed, with the aim of promoting consistency and minimising technique-related variability.

The importance of a placebo group when assessing responsiveness was highlighted by Benito et al. (14) in their evaluation of the Feline Musculoskeletal Pain Index (FMPI), where both placebo- and meloxicam-treated cats showed significant within-group reductions in pain scores. However, logistic regression analysis failed to detect a difference between treatment groups, leading the authors to question the scale’s responsiveness to intervention despite apparent clinical improvement. The use of a placebo group was pivotal in revealing this limitation and subsequently enabled the refinement of the FMPI into a shorter form (FMPI-sf) (43). In the present study, inclusion of a control or placebo group was not feasible due to the retrospective nature of the video dataset, which was derived from routine clinical evaluations at the pain management unit. In this context, withholding analgesia from dogs with suspected pain would have contravened welfare principles and clinical standards. While the observed score reductions following intervention suggest the LSPain Scale responsiveness, the absence of a control group limits the ability to attribute these changes solely to the analgesic intervention. The potential influence of natural variation, regression to the mean, or caregiver placebo effects cannot be fully excluded (14, 44, 45).

Further confirmation of responsiveness under controlled conditions would be valuable, using ethically acceptable alternatives to placebo, such as delayed-treatment designs or add-on trials (46). Nonetheless, based on the authors’ clinical experience, the analgesic interventions used in this cohort resulted in clear and consistent clinical improvement, both in these cases and more broadly in practice.

The LSPain Scale was developed to grade pain expression during clinical examination, without aiming to determine the anatomical source of pain. In clinical practice, when a dog displays discomfort on LS palpation, localising the primary pain generator can be challenging. This is particularly relevant given the frequent coexistence of hip and LS pathology in dogs and the overlap in their clinical signs (1). Furthermore, as reported in our previous MRI-based study, LS pain may still be present in dogs without detectable structural abnormalities, highlighting the limitations of imaging-based diagnosis in certain cases (1). To minimise diagnostic confounding during validation, dogs with concurrent hip pathology were excluded. This approach aimed to ensure that pain behaviours observed during palpation could be more confidently attributed to the LS region. As previously highlighted in the literature, a clinical instrument should be applied as originally described and validated to preserve its measurement properties (42). Although the LSPain Scale was developed in a cohort of dogs with confirmed LS pathology, it is not intended to diagnose the underlying cause of pain. Instead, it offers a standardised method to quantify behavioural responses to LS palpation. In clinical practice, the scale may aid in recognising pain localised to this region and support subsequent diagnostic or therapeutic decisions, regardless of the underlying aetiology.

The development of small animal pain assessment scales has typically involved input from multiple individuals, including veterinary professionals and caregivers. For instance, the Glasgow Composite Measure Pain Scale (CMPS) was developed through a multi-phase process involving hundreds of veterinary professionals and a core team of veterinary researchers. Its short-form version was subsequently derived by restructuring and refining the original scale to enhance clinical utility. This process included incorporation of feedback from over 500 practising veterinary surgeons during pre-testing, alongside insights from previous work (25). The CBPI was developed by a group of 10 veterinarians in collaboration with many dog caregivers (15). The Feline Musculoskeletal Pain Index (FMPI) was conceived via multiple stages involving a team of veterinary researchers and cat owners (14). The items of the Feline Grimace Scale (FGS) were conceived by two experienced clinicians, and the FGS was subsequently validated by four independent observers (13). In contrast, the LSPain Scale was developed by a single clinician. This highlights the variability in scale development processes, which may involve large multidisciplinary teams or be carried out by smaller groups or individual clinicians. In the case of the present study, this approach may have inherently introduced a degree of bias towards individual clinical experience, the scale’s structure was grounded in established principles of behavioural pain assessment (12) and supported by the co-authors in its final format prior to prospective observer evaluation. Despite its individual origin, the LSPain Scale demonstrated promising construct validity and clinical applicability in this initial validation. As with other observer-based veterinary clinical metrology instruments that have undergone refinement over time (12, 43), future amendments to the LSPain Scale may be considered as clinical experience accumulates. The authors are open to further discussion and collaboration regarding potential improvements of this scale. However, any modifications, whether proposed by the original developers or by others, should be followed by formal revalidation to ensure the scale’s psychometric integrity is maintained (42).

In conclusion, the LSPain Scale is a simple, valid, and reliable clinician-based instrument for assessing LS pain in dogs. It provides a structured behavioural framework that complements caregiver-reported tools and supports clinical decision-making during diagnosis, monitoring, and treatment. This initial validation represents a meaningful step towards the development of standardised, condition-specific tools to improve the assessment and management of canine LS pain in veterinary practice.

## Data Availability

The original contributions presented in the study are included in the article/Supplementary material, further inquiries can be directed to the corresponding author.

## Footnotes

^1^ Randomizer.org

## Supplementary material

SUPPLEMENTARY VIDEO 1 Training video to support standardised application of the LSPain Scale.

SUPPLEMENTARY VIDEO 2 Training video to support standardised application of the LSPain Scale – English captions.

SUPPLEMENTARY DATA SHEET 1 Anonymised dataset (GDPR compliant) supporting the results of this study.

## References

[1] Medina-Serra R López-Abradelo P Belda E Riding-Medina H Laredo FG Marwood R. Multivariable analysis of the association between lumbar and lumbosacral MRI-diagnosed spinal pathologies and pain in dogs. Animals. (2025) 15:761. doi: 10.3390/ani15050761, PMID: 40076044 PMC11898813

[2] Lascelles BDX Brown DC Conzemius MG Gill M Oshinsky ML Sharkey M. Measurement of chronic pain in companion animals: discussions from the pain in animals workshop (PAW) 2017. Vet J. (2019) 250:71–8. doi: 10.1016/j.tvjl.2019.07.001, PMID: 31383423

[3] Brown DC Coetzee J Gill M Johnson C Mohapatra DP Oshinsky ML. Outcome assessment in veterinary pain studies: a pain in animals workshop (PAW) perspective. Frontiers in Pain Research. (2025) 6:1579155. doi: 10.3389/fpain.2025.1579155, PMID: 40276168 PMC12018479

[4] Gomes SA Lowrie M Targett M. Single dose epidural methylprednisolone as a treatment and predictor of outcome following subsequent decompressive surgery in degenerative lumbosacral stenosis with foraminal stenosis. Vet J. (2020) 257:105451. doi: 10.1016/j.tvjl.2020.105451, PMID: 32546351

[5] Janssens LA Beosier Y Daems R. Lumbosacral degenerative stenosis in the dog: the results of epidural infiltration with methylprednisolone acetate: a retrospective study. Vet Comp Orthop Traumatol. (2009) 22:486–91. doi: 10.3415/VCOT-08-07-0055, PMID: 19876516

[6] Brown DC Boston RC Coyne JC Farrar JT. Ability of the canine brief pain inventory to detect response to treatment in dogs with osteoarthritis. J Am Vet Med Assoc. (2008) 233:1278–83. doi: 10.2460/javma.233.8.1278, PMID: 19180716 PMC2896492

[7] Brown DC Boston R Coyne JC Farrar JT. A novel approach to the use of animals in studies of pain: validation of the canine brief pain inventory in canine bone Cancer. Pain Med. (2009) 10:133–42. doi: 10.1111/j.1526-4637.2008.00513.x, PMID: 18823385 PMC2644730

[8] Lascelles BDX Knazovicky D Case B Freire M Innes JF Drew AC. A canine-specific anti-nerve growth factor antibody alleviates pain and improves mobility and function in dogs with degenerative joint disease-associated pain. BMC Vet Res. (2015) 11:101. doi: 10.1186/s12917-015-0413-x, PMID: 25926287 PMC4419463

[9] Ruel HLM Watanabe R Evangelista MC Beauchamp G Auger J-P Segura M. Pain burden, sensory profile and inflammatory cytokines of dogs with naturally-occurring neuropathic pain treated with gabapentin alone or with meloxicam. PLoS One. (2020) 15:e0237121. doi: 10.1371/journal.pone.0237121, PMID: 33253197 PMC7703878

[10] DeVellis R. Scale development: theory and applications. 4th ed. Thousand Oaks, CA: SAGE Publications (2017).

[11] Martin P Bateson P. Measuring behaviour. Cambridge: Cambridge University Press (2007).

[12] Reid J Nolan A Hughes J Lascelles D Pawson P Scott E. Development of the short-form Glasgow composite measure pain scale (CMPS-SF) and derivation of an analgesic intervention score. Anim Welf. (2007) 16:97–104. doi: 10.1017/S096272860003178X

[13] Evangelista MC Watanabe R Leung VSY Monteiro BP O’Toole E Pang DSJ. Facial expressions of pain in cats: the development and validation of a feline grimace scale. Sci Rep. (2019) 9:19128. doi: 10.1038/s41598-019-55693-8, PMID: 31836868 PMC6911058

[14] Benito J Hansen B DePuy V Davidson GS Thomson A Simpson W. Feline musculoskeletal pain index: responsiveness and testing of criterion validity. J Vet Intern Med. (2013) 27:474–82. doi: 10.1111/jvim.12077, PMID: 23551140

[15] Brown DC Boston RC Coyne JC Farrar JT. Development and psychometric testing of an instrument designed to measure chronic pain in dogs with osteoarthritis. Am J Vet Res. (2007) 68:631–7. doi: 10.2460/ajvr.68.6.631, PMID: 17542696 PMC2907349

[16] Robinson CL Phung A Dominguez M Remotti E Ricciardelli R Momah DU. Pain scales: what are they and what do they mean. Curr Pain Headache Rep. (2024) 28:11–25. doi: 10.1007/s11916-023-01195-2, PMID: 38060102

[17] Brondani JT Luna SPL Padovani CR. Refinement and initial validation of a multidimensional composite scale for use in assessing acute postoperative pain in cats. Am J Vet Res. (2011) 72:174–83. doi: 10.2460/ajvr.72.2.174, PMID: 21281191

[18] Boateng GO Neilands TB Frongillo EA Melgar-Quiñonez HR Young SL. Best practices for developing and validating scales for health, social, and behavioral research: a primer. Front Public Health. (2018) 6:149. doi: 10.3389/fpubh.2018.00149, PMID: 29942800 PMC6004510

[19] Walton MB Cowderoy E Lascelles D Innes JF. Evaluation of construct and criterion validity for the ‘Liverpool osteoarthritis in dogs’ (LOAD) clinical metrology instrument and comparison to two other instruments. PLoS One. (2013) 8:e58125. doi: 10.1371/journal.pone.0058125, PMID: 23505459 PMC3591443

[20] Koo TK Li MY. A guideline of selecting and reporting intraclass correlation coefficients for reliability research. J Chiropr Med. (2016) 15:155–63. doi: 10.1016/j.jcm.2016.02.012, PMID: 27330520 PMC4913118

[21] Mota-Rojas D Whittaker AL Coria-Avila GA Martínez-Burnes J Mora-Medina P Domínguez-Oliva A. How facial expressions reveal acute pain in domestic animals with facial pain scales as a diagnostic tool. Front Vet Sci. (2025) 12:1546719. doi: 10.3389/fvets.2025.1546719, PMID: 40104548 PMC11913824

[22] Rojas DM Velarde A Broom DM Orihuela A. ANIMAL WELFARE BOOK. Chapter 10. Animal welfare and the neurobiological basis of pain / El Bienestar animal y las bases Neurobiológicas Del dolor. In: BM Editors. Animal welfare book. Mexico (2024). 331–58.

[23] Warden V Hurley AC Volicer L. Development and psychometric evaluation of the pain assessment in advanced dementia (PAINAD) scale. J Am Med Dir Assoc. (2003) 4:9–15. doi: 10.1097/01.JAM.0000043422.31640.F7, PMID: 12807591

[24] Shipley H Guedes A Graham L Goudie-DeAngelis E Wendt-Hornickle E. Preliminary appraisal of the reliability and validity of the Colorado State University feline acute pain scale. J Feline Med Surg. (2019) 21:335–9. doi: 10.1177/1098612X18777506, PMID: 29848148 PMC10814628

[25] Holton L Pawson P Nolan A Reid J Scott EM. Development of a behaviour-based scale to measure acute pain in dogs. Vet Rec. (2001) 148:525–31. doi: 10.1136/vr.148.17.525, PMID: 11354645

[26] Cachon T Frykman O Innes JF Lascelles BDX Okumura M Sousa P. Face validity of a proposed tool for staging canine osteoarthritis: canine OsteoArthritis staging tool (COAST). Vet J. (2018) 235:1–8. doi: 10.1016/j.tvjl.2018.02.017, PMID: 29704933

[27] Stabile M Van Ryssen B Minei S Coppieters E Crovace A Lacitignola L. Observational study of the clinical value of the canine osteoarthritis staging tool. Vet J. (2022) 283–284:105832. doi: 10.1016/j.tvjl.2022.105832, PMID: 35487477

[28] Wells JR Young AL Crane A Moyaert H Michels G Wright A. Linguistic validation of the canine brief pain inventory (CBPI) for global use. Front Vet Sci. (2021) 8:769112. doi: 10.3389/fvets.2021.769112, PMID: 34912877 PMC8666957

[29] Olcoz M Cabezas MÁ Rocca GD Gómez de Segura IA. Translation to Spanish and linguistic validation of the canine brief pain inventory. Front Vet Sci. (2023) 10:1203453. doi: 10.3389/fvets.2023.1203453, PMID: 37456964 PMC10348357

[30] Miró J de la Vega R Solé E Racine M Jensen MP Gálan S. Defining mild, moderate, and severe pain in Young people with physical disabilities. Disabil Rehabil. (2017) 39:1131–5. doi: 10.1080/09638288.2016.1185469, PMID: 27291566 PMC5553452

[31] Treede R-D Rief W Barke A Aziz Q Bennett MI Benoliel R. Chronic pain as a symptom or a disease: the IASP classification of chronic pain for the international classification of diseases (ICD-11). Pain. (2019) 160:19–27. doi: 10.1097/j.pain.0000000000001384, PMID: 30586067

[32] Von Korff M DeBar LL Krebs EE Kerns RD Deyo RA Keefe FJ. Graded chronic pain scale revised: mild, bothersome, and high-impact chronic pain. Pain. (2020) 161:651–61. doi: 10.1097/j.pain.0000000000001758, PMID: 31764390 PMC7097879

[33] Taub C Gordon KS Goulet J Lee A Mayhew M Von Korff M. Graded chronic pain scale revised: validation in a veteran sample. Pain Med. (2023) 24:1169–75. doi: 10.1093/pm/pnad068, PMID: 37220899 PMC10546477

[34] WHO Expert Committee Cancer Pain Relief and Palliative Care. Report of a WHO expert committee. Geneva: World Health Organization Technical Reports Series (1990), 804, 1–75. 1702248

[35] Boonstra AM Schiphorst Preuper HR Balk GA Stewart RE. Cut-off points for mild, moderate, and severe pain on the visual analogue scale for pain in patients with chronic musculoskeletal pain. Pain. (2014) 155:2545–50. doi: 10.1016/j.pain.2014.09.014, PMID: 25239073

[36] Boonstra AM Stewart RE Köke AJA Oosterwijk RFA Swaan JL Schreurs KMG. Cut-off points for mild, moderate, and severe pain on the numeric rating scale for pain in patients with chronic musculoskeletal pain: variability and influence of sex and catastrophizing. Front Psychol. (2016) 7:1466. doi: 10.3389/fpsyg.2016.01466, PMID: 27746750 PMC5043012

[37] Moody CM Niel L Pang DJ. Is training necessary for efficacious use of the Glasgow feline composite measure pain scale? Can Vet J. (2022) 63:609–16. 35656525 PMC9112359

[38] Zhang EQ Leung VS Pang DS. Influence of rater training on inter- and Intrarater reliability when using the rat grimace scale. J Am Assoc Lab Anim Sci. (2019) 58:178–83. doi: 10.30802/AALAS-JAALAS-18-000044, PMID: 30755291 PMC6433356

[39] Adami C Filipas M John C Skews K Dobson E. Inter-observer reliability of three feline pain scales used in clinical practice. J Feline Med Surg. (2023) 25:1098612X231194423. doi: 10.1177/1098612X231194423, PMID: 37747309 PMC10812030

[40] Della Rocca G Colpo R Reid J Di Salvo A Scott M. Creation and validation of the Italian version of the Glasgow composite measure pain scale-short form (ICMPS-SF). Vet Ital. (2018) 54:251–60. doi: 10.12834/VetIt.699.3421.3, PMID: 30575003

[41] Ask K Andersen PH Tamminen L-M Rhodin M Hernlund E. Performance of four equine pain scales and their association to movement asymmetry in horses with induced orthopedic pain. Front Vet Sci. (2022) 9:938022. doi: 10.3389/fvets.2022.938022, PMID: 36032285 PMC9411665

[42] Testa B Reid J Scott ME Murison PJ Bell AM. The short form of the Glasgow composite measure pain scale in post-operative analgesia studies in dogs: a scoping review. Front Vet Sci. (2021) 8:751949. doi: 10.3389/fvets.2021.751949, PMID: 34660773 PMC8515184

[43] Enomoto M Lascelles BDX Robertson JB Gruen ME. Refinement of the feline musculoskeletal pain index (FMPI) and development of the short-form FMPI. J Feline Med Surg. (2022) 24:142–51. doi: 10.1177/1098612X211011984, PMID: 34002643 PMC10812168

[44] Conzemius MG Evans RB. Caregiver placebo effect for dogs with lameness from osteoarthritis. J Am Vet Med Assoc. (2012) 241:1314–9. doi: 10.2460/javma.241.10.1314, PMID: 23113523

[45] Gruen ME Dorman DC Lascelles BDX. Caregiver placebo effect in analgesic clinical trials for cats with naturally occurring degenerative joint disease-associated pain. Vet Rec. (2017) 180:473–3. doi: 10.1136/vr.104168, PMID: 28270539 PMC5498173

[46] Streiner DL. Alternatives to placebo-controlled trials. Can J Neurol Sci. (2007) 34:S37–41. doi: 10.1017/S0317167100005540 17469680

